# Accidents and undetermined deaths: re-evaluation of nationwide samples from the Scandinavian countries

**DOI:** 10.1186/s12889-016-3135-5

**Published:** 2016-05-27

**Authors:** Ingvild Maria Tøllefsen, Ingemar Thiblin, Karin Helweg-Larsen, Erlend Hem, Marianne Kastrup, Ullakarin Nyberg, Sidsel Rogde, Per-Henrik Zahl, Gunvor Østevold, Øivind Ekeberg

**Affiliations:** Department of Acute Medicine, Oslo University Hospital Ullevaal, Box 4950, Nydalen, N-0424 Oslo Norway; Department of Behavioural Sciences in Medicine, Institute of Basic Medical Sciences, Faculty of Medicine, University of Oslo, Box 1072, Blindern, N-0316 Oslo Norway; Department of Surgical Sciences, Uppsala University, Box 256, 751 05 Uppsala, Sweden; Department of Social Medicine and Public Health Research, Copenhagen University, Nørregade 10, Copenhagen K, DK-1165 Denmark; Division of Mental Health and Addiction, Oslo University Hospital Ullevaal, Box 4950, Nydalen, N-0424 Oslo Norway; Amalievej 23, Frederiksberg, DK 1875 Denmark; Stockholm Centre for Psychiatric Research and Education, Department of Clinical Neuroscience, Karolinska Institutet, Sweden, Norra Stocholms psychiatri S:t Görans sjukhus, Stockholm, SWE-112 81 Sweden; Norwegian Institute of Public Health, Box 4404, Nydalen, N-0403 Oslo Norway; Institute of Clinical Medicine, University of Oslo, Box 1072, Blindern, N- 0316 Oslo Norway; Division of Medicine, Department of Acute Medicine, Oslo University Hospital Ullevaal, Box 4950, Nydalen, N-0424 Oslo Norway

**Keywords:** Accidents, Autopsy, Reclassification, Suicide statistics, Undetermined deaths

## Abstract

**Background:**

National mortality statistics should be comparable between countries that use the World Health Organization’s International Classification of Diseases. Distinguishing between manners of death, especially suicides and accidents, is a challenge. Knowledge about accidents is important in prevention of both accidents and suicides. The aim of the present study was to assess the reliability of classifying deaths as accidents and undetermined manner of deaths in the three Scandinavian countries and to compare cross-national differences.

**Methods:**

The cause of death registers in Norway, Sweden and Denmark provided data from 2008 for samples of 600 deaths from each country, of which 200 were registered as suicides, 200 as accidents or undetermined manner of deaths and 200 as natural deaths. The information given to the eight experts was identical to the information used by the Cause of Death Register. This included death certificates, and if available external post-mortem examinations, forensic autopsy reports and police reports.

**Results:**

In total, 69 % (Sweden and Norway) and 78 % (Denmark) of deaths registered in the official mortality statistics as accidents were confirmed by the experts. In the majority of the cases where disagreement was seen, the experts reclassified accidents to undetermined manner of death, in 26, 25 and 19 % of cases, respectively. Few cases were reclassified as suicides or natural deaths. Among the extracted accidents, the experts agreed least with the official mortality statistics concerning drowning and poisoning accidents. They also reported most uncertainty in these categories of accidents. In a second re-evaluation, where more information was made available, the Norwegian psychiatrist and forensic pathologist increased their agreement with the official mortality statistics from 76 to 87 %, and from 85 to 88 %, respectively, regarding the Norwegian and Swedish datasets. Among the extracted undetermined deaths in the Swedish dataset, the two experts reclassified 22 and 51 %, respectively, to accidents.

**Conclusion:**

There was moderate agreement in reclassification of accidents between the official mortality statistics and the experts. In the majority of cases where there was disagreement, accidents were reclassified as undetermined manner of death, and only a small proportion as suicides.

## Background

In mortality statistics in European and many non-European countries, the cause of death is classified according to the World Health Organization’s (WHO) International Statistical Classification of Diseases and Related Health Problems (ICD), and thus these countries should have comparable mortality statistics. The three Scandinavian countries, Denmark, Norway and Sweden have used the 10th edition of ICD (ICD-10) since 1994, 1996 and 1997, respectively. This enables comparisons between the Scandinavian countries. Researchers frequently compare mortality statistics over time and between countries, to develop policies and preventive measures. Therefore, in-depth knowledge of the procedures for obtaining mortality statistics is necessary. A number of factors affect the comparability of mortality statistics over time. The most important factors are changes in routines in collection of mortality data, changes in diagnostic methods and medical terms and changed rules for classifying the underlying cause of death.

Under-reporting of suicide deaths is a debated issue, and studies have reported as much as a fourfold difference between the official suicide rate and the reclassified rate. Suicides are misclassified as accidental drowning, accidental poisoning, traffic accidents, “ill-defined and unknown cause of mortality”, or “undetermined intent” [1–8]. Regarding suicide statistics, different confounding variables are important: governmental sanctions, as suicide is still a criminal offence in some countries [9], religious sanctions [10], insurance considerations, the social position of the deceased [11], and differences in attitude of coroners, medical examiners or physicians regarding whether suicide is the manner of death: “beyond reasonable doubt” in contrast to the “balance of probability” [12].

Determining the manner of death (i.e., suicide, accident, homicide, undetermined or natural death) could be a challenge in some cases, and few studies have evaluated the manner of death within a country or between countries [13]. Classifications as accidents and undetermined manner of death probably contribute the most to possible “missed” suicides. Hence, knowledge about accidents and undetermined manner of death is important in prevention of both accidents and suicides. To our knowledge, no previous studies have compared cross-national differences between undetermined death and certain categories of accidents regarding misclassification and certainty in diagnoses.

The aim of the present study was to assess the reliability of classifying deaths as accidents and undetermined manner of deaths in the three Scandinavian countries and to compare cross-national differences.

## Methods

### Description of data

The reclassification was based on 1800 deaths in 2008 among people aged 18 years or older, 600 from each of the three Scandinavian countries. In the national Cause of Death Register in each country, 200 of these deaths were registered as suicides, 200 as accidents or undetermined manner of deaths, and 200 as natural deaths by different causes. The sample of 200 suicides included all suicide methods (ICD-10: X60–X84, Y870). The sample of 200 accidents and undetermined manner of deaths included traffic accidents (ICD-10: V01–V99), accidental poisoning (ICD-10: X40–X49), accidental drowning (ICD-10: W65–W74), accidental fire and flame (ICD-10: X00–X09) and undetermined intent (ICD-10: Y10–Y34, Y872). The Norwegian and Swedish accident samples did not include all types of traffic accidents but comprised the category “car occupant injured in transport accident” (ICD-10: V43–V45.5, V47–V48.5, V49.4), which was most likely to include “missed suicides”. The Danish dataset included all types of traffic accidents (pedestrian, pedal cyclist, bus occupant and so on.) (ICD-10: V01–V99). The Swedish dataset also included deaths registered as undetermined intent. The Norwegian dataset included no cases of undetermined intent because no cases were coded as undetermined intent in the Norwegian Cause of Death Register in 2008. In all three countries, natural deaths included deaths registered with a psychiatric disorder as the underlying cause of death. This selection of natural deaths was made because of the reported higher risk of suicide for people with these mental disorders [14]. These included “mental and behavioural disorders due to psychoactive substance use” (ICD-10: F10–F19), “schizophrenia, schizotypal and delusional disorders” (ICD-10: F20–F29), “mood (affective) disorders” (ICD-10: F30–F39) and “disorders of adult personality and behaviour” (ICD-10: F60–F69). The Danish dataset also included “ill-defined and unknown causes of mortality” (ICD-10: R96–R99) (Table 1).

**Table 1:** Overview of the cases extracted from the cause of death registers in Scandinavia

Manner and cause of death (ICD-10 codes)	Norway	Sweden	Denmark
	n	n	n
Suicides (X60–84, Y87.0)	200	200	200
Accidents	200	200	199
Traffic accidents (V01–99)^a^	29	34	45
Accidental poisoning (X40–49)	129	70	104
Accidental drowning (W65–74)	16	21	21
Accidental fire and flame (X00–09)	26	15	29^b^
Undetermined intent (Y10–34, Y87.2)	0	60	0
Natural deaths	200	200	200
Mental and behavioural disorders due to psychoactive substance use (F10–19)	155	149	59
Schizophrenia, schizotypal and delusional disorders (F20–29)	19	14	31
Mood (affective) disorders (F30–39)	24	37	51
Disorders of adult personality and behaviour (F60–69)	2	0	0
Ill-defined and unknown causes of mortality (R96–99)	0	0	59
Total number of cases	600	600	599

^a^The Norwegian and Swedish datasets included a selection of traffic accidents (ICD-10: V43–45.5, V47–48.5, V49.4), while the Danish dataset included all traffic accidents (ICD-10: V01–99 (V01–99). In the Danish dataset, 14 cases were within the same selection of traffic accidents as in the Norwegian and Swedish datasets (i.e., V43–45.5, V47–48.5, V49.4)

^b^One male was excluded because of age under 18 years

The reclassification was based on information given on death certificates and autopsy reports. In the Norwegian sample of 600 deaths, autopsies had been performed on 325 (54 %), and 86 of these had a complete autopsy report available. In a further 239 cases, the reclassification was based on information on the manner and cause of death from a revised death certificate and the results from the autopsy were documented using the specific coding Systematized Nomenclature of Medicine (SNOMED). SNOMED is a systematic, computer-processable collection of clinical terminology used by pathologists. Local regulations and practices explain why some pathology departments submit a complete autopsy report, while others only send a revised death certificate and SNOMED codes to the Norwegian Cause of Death Register. The information given to the experts was identical to the information used by the Cause of Death Register. The Swedish dataset did not include any autopsy reports, even though an autopsy had been performed in 483 (81 %) of the 600 cases. In cases of an unnatural manner of death in the Danish dataset, the death certificates contained in text form an excerpt of the clinical data, information from a death scene investigation, findings of an external post-mortem examination and an autopsy report when available. In 191 (32 %) of the 600 Danish cases, an autopsy had been performed and information from the autopsy report was included in the death certificates.

### Re-evaluations of the data sets

All cases were de-identified and given a random identification number before they were individually re-evaluated by the experts, although the age and sex were indicated on the death certificates. The Death certificates included the marked or written manners and/or causes of death set by the certifying physician, but did not include the ICD-10-code set by the Cause of Death Register. The eight experts from the three Scandinavian countries who performed the reclassifications were psychiatrists (ØE, MK, UN), forensic pathologists (SR, KHL, IT) and expert coders (GØ, OBL). The expert coders have in-depth knowledge about implementing the WHO’s ICD principles and were thus able to evaluate the reliability of the coding systems. The forensic pathologists have special competence in the evaluation of the manner and cause of death. The psychiatrists’ skills include assessing motives behind human behaviour.

**Fig. 1:**
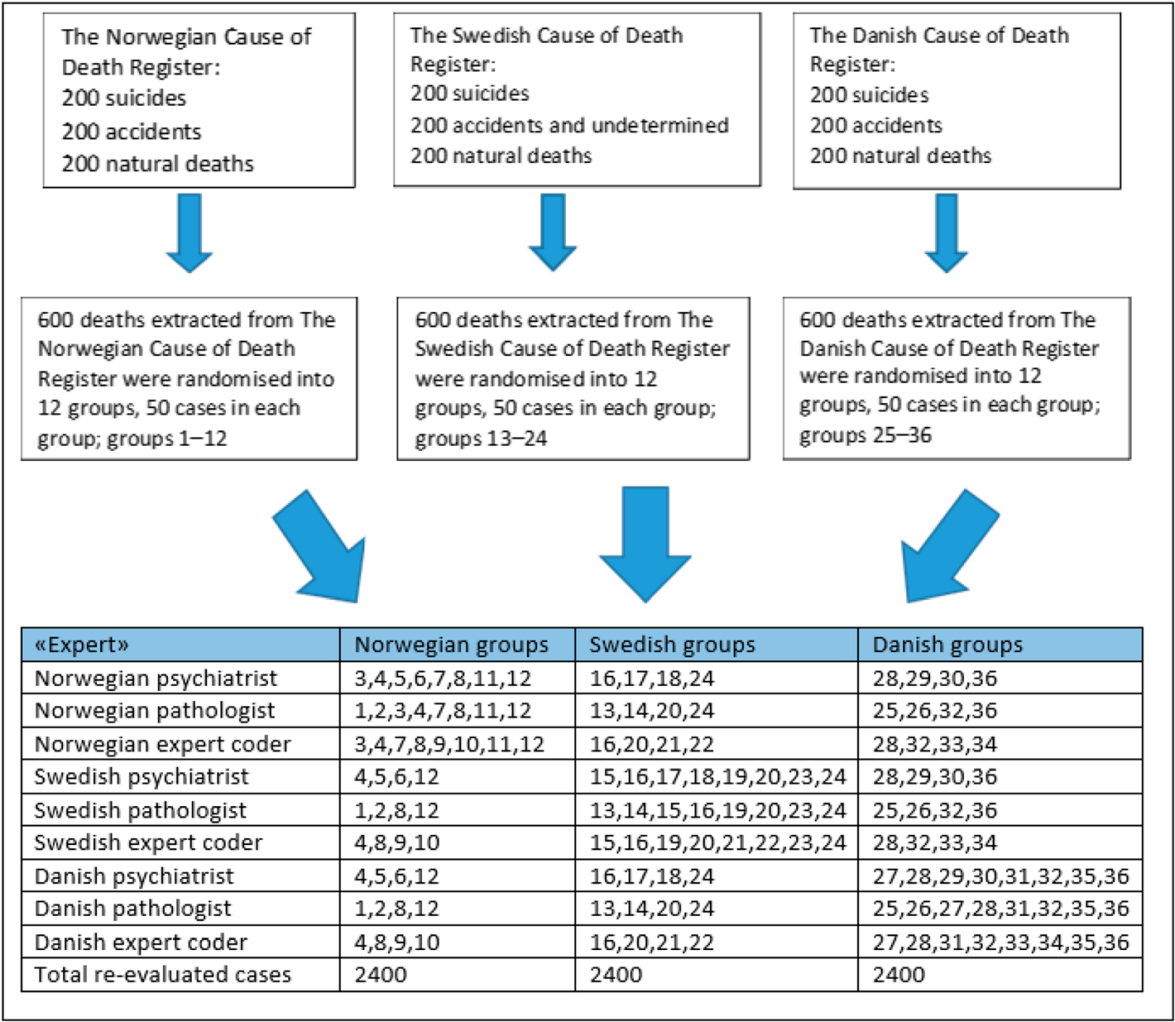
Per protocol distribution of the extracted cases

Per protocol, we divided the 600 cases from each country into 12 groups of 50 cases to ensure the reclassification of cases from all three countries by all three expert groups. In each group, a random sample of causes of death was included. Thus, all 1800 cases were re-evaluated by at least three experts, and some cases were re-evaluated by all experts (Fig. 1). In the present study, the Norwegian psychiatrist re-evaluated 1000 cases (600 from the Norwegian dataset and 200 from each of the Swedish and Danish sets). The Norwegian and Swedish forensic pathologists and the Norwegian expert coder re-evaluated 800 cases (400 deaths from his/her own country and 200 from each of the other two). The Swedish psychiatrist and the three Danish experts re-evaluated 600 cases each, 200 from each country.

For each case, a coding form was used to assess the manner of death (i.e., natural death, suicide, accident, homicide or undetermined) and then the cause of death (hanging, cardio-vascular disease, etc.). The expert then stated the level of certainty regarding the manner and cause of death as follows: 1, certain; 2, possible; 3, uncertain; 4, insufficient information to determine the manner and cause of death; and 5, insufficient information to determine the cause of death. In the further processing of data, certainty group levels 1 and 2 were merged into one group (certain), group level 3 (uncertain) was unchanged and group levels 4 and 5 were merged into another group (insufficient information).

### Second re-evaluation of the Norwegian and Swedish data sets

Because much of the information was sparse in the Norwegian and Swedish cases, two of the experts (the Norwegian psychiatrist and forensic pathologist) did a second reclassification of Norwegian cases for which a forensic autopsy report had been made available and, in Swedish cases, for which a forensic autopsy and police report had been made available. This included 180 Norwegian and 483 Swedish cases. In the Norwegian dataset, in addition to these 180 cases, 59 cases contained a death certificate issued by a forensic pathologist plus SNOMED codes. These cases included autopsies performed in institutions other than the Norwegian Institute of Public Health and were not included in the second re-evaluation. The 59 deaths where the autopsies were made in institutions other than the Norwegian Institute of Public Health were not included because it would have taken a lot of time to get permission to extract autopsy reports from all of these institutions. The Norwegian psychiatrist reclassified the same cases as in the first re-evaluation in which an autopsy report were made available. The Norwegian psychiatrist re-evaluated all 180 Norwegian cases and the Norwegian forensic pathologist 124 in the second re-evaluation. The Norwegian psychiatrist and forensic pathologist also re-evaluated the same 200 Swedish cases as in the first re-evaluation and all cases classified as undetermined intent in the Swedish Cause of Death Register. In total, the Norwegian psychiatrist re-evaluated 235 and the Norwegian forensic pathologist re-evaluated 247 Swedish cases in the second re-evaluation.

### Statistical analysis

SPSS statistics 21.0 (Armonk, NY) was used for data analysis. Differences in demographic characteristics and level of certainty were analysed using chi-squared tests with a significance level of 0.05. Crosstabs were used to compare official statistics with the re-evaluations. Cohen’s kappa was used to assess inter-rater agreement between the experts. All cases were plotted manually in SPSS, and in a control analysis of 800 numbers, there were 0.6 % incorrectly plotted numbers.

### Ethics approval

This study was approved by the Regional Committees for Medical and Health Research Ethics, South East Norway. In addition, the Norwegian Institute of Public Health, the Higher Prosecuting Authority in Norway and Oslo University Hospital Data Inspectorate approved the study. The National Board of Forensic Medicine and the National Board of Health and Welfare in Sweden and in Denmark also approved the study.

## Results

### Description of sample

A forensic autopsy had been performed in 998 (55 %) of the 1800 cases, varying from 32 % in the Danish dataset to 81 % in the Swedish dataset (Table 2). For all three countries, natural deaths had the lowest proportion of autopsies, and accidents the highest proportion in the Norwegian and Danish dataset. Suicides and undetermined deaths had the highest proportion of autopsies in the Swedish dataset. For intentional self-poisoning, the proportions of autopsies were 97 % (Sweden), 77 % (Norway) and 34 % (Denmark). For accidental poisoning, the proportions of autopsies were 89 % (Sweden), 87 % (Norway) and 95 % (Denmark). When the total sample was divided into age groups, there were significantly (*p* < 0.001) more autopsies in the younger age groups: aged 18–50 (74 %), aged 51–70 (56 %), above 71 years (24 %). This difference was also significant (*p* < 0.001) for each of the three datasets. There was no significant difference in the proportion of autopsies between males and females.

**Table 2:** Demographic characteristics; autopsy reports by country, manner of death, age and gender

Autopsies by manner of death	Total	Aged 18–50	Aged 51–70	Aged ≥71	Pearson Chi-Squared (*χ* ^2^)
Norwegian dataset (Nor)					
Number of cases (n)	600	283 (47 %)	197 (33 %)	120 (20 %)	
Autopsies	325 (54 %)	208 (74 %)	87 (44 %)	30 (25 %)	<0.001
Male gender (%)	432 (72 %)				0.234
Natural deaths (F10–39, F60–69)	32 (16 %)	11 (34 %)	19 (18 %)	2 (3 %)	<0.001
Suicides (X60–84, Y870)	136 (68 %)	84 (71 %)	39 (67 %)	13 (54 %)	0.262
Accidents (V43–45.5, V47–48.5, V49.4, X40–49, W65–74, X00–09)	157 (79 %)	113 (85 %)	29 (83 %)	15 (47 %)	<0.001
Swedish dataset (Swe)					
Number of cases (n)	600	227 (38 %)	239 (40 %)	134 (22 %)	
Autopsies	483 (81 %)	214 (94 %)	208 (87 %)	61 (46 %)	<0.001
Male gender (%)	403 (67 %)				0.096
Natural deaths (F10–39, F60–69)	108 (54 %)	25 (86 %)	77 (76 %)	6 (9 %)	<0.001
Suicides (X60–84, Y870)	192 (96 %)	91 (97 %)	76 (96 %)	25 (93 %)	0.611
Accidents (V43–45.5, V47–48.5, V49.4, X40–49, W65–74, X00–09)	124 (89 %)	65 (92 %)	36 (92 %)	23 (77 %)	0.069
Undetermined deaths (Y10–34, Y872)	59 (98 %)	33 (100 %)	19 (95 %)	7 (100 %)	0.362
Danish dataset (Dan)					
Number of cases (n)	599	232 (39 %)	183 (30 %)	184 (31 %)	
Autopsies	190 (32 %)	124 (53 %)	50 (27 %)	16 (9 %)	<0.001
Male gender (%)	388 (65 %)				0.879
Natural deaths (F10–39, F60–69, R96–99)	15 (8 %)	7 (37 %)	7 (10 %)	1 (1 %)	<0.001
Suicides (X60–84, Y870)	36 (18 %)	22 (25 %)	14 (20 %)	0	0.003
Accidents (V01–99, X40–49, W65–74, X00–09)	139 (70 %)	96 (77 %)	29 (66 %)	15 (48 %)	0.007
	Nor, n (%)	Swe, n (%)	Dan, n (%)	Pearson Chi-Squared (*χ* ^2^)	
Car occupant injured in transport accidents (V43–V45.5, V47–V48.5, V49.4)	17 (59 %)	32 (94 %)	4 (29 %)	<0.001	
Transport accidents (V01–V99)			12 (27 %)		
Accidental poisonings (X40–49)	112 (87 %)	62 (89 %)	99 (95 %)	0.093	
Accidental drownings (W65–74)	9 (56 %)	16 (76 %)	9 (43 %)	0.088	
Accidental exposure to smoke, fire and flames (X00–09)	19 (73 %)	14 (93 %)	19 (66 %)	0.149	
Event of undetermined intent (Y10–34, Y872)		59 (98 %)			

Cases in which an autopsy was performed according to country, manner of death, age and gender. The lowermost part of the table presents transport, poisoning, drowning and fire accidents, in addition to cases in which the manner of death was undetermined. Percentages are given in parentheses. To analyse differences in the number of autopsies between age groups and countries, chi-squared tests were used

### Reclassification of the datasets

**Fig. 2:**
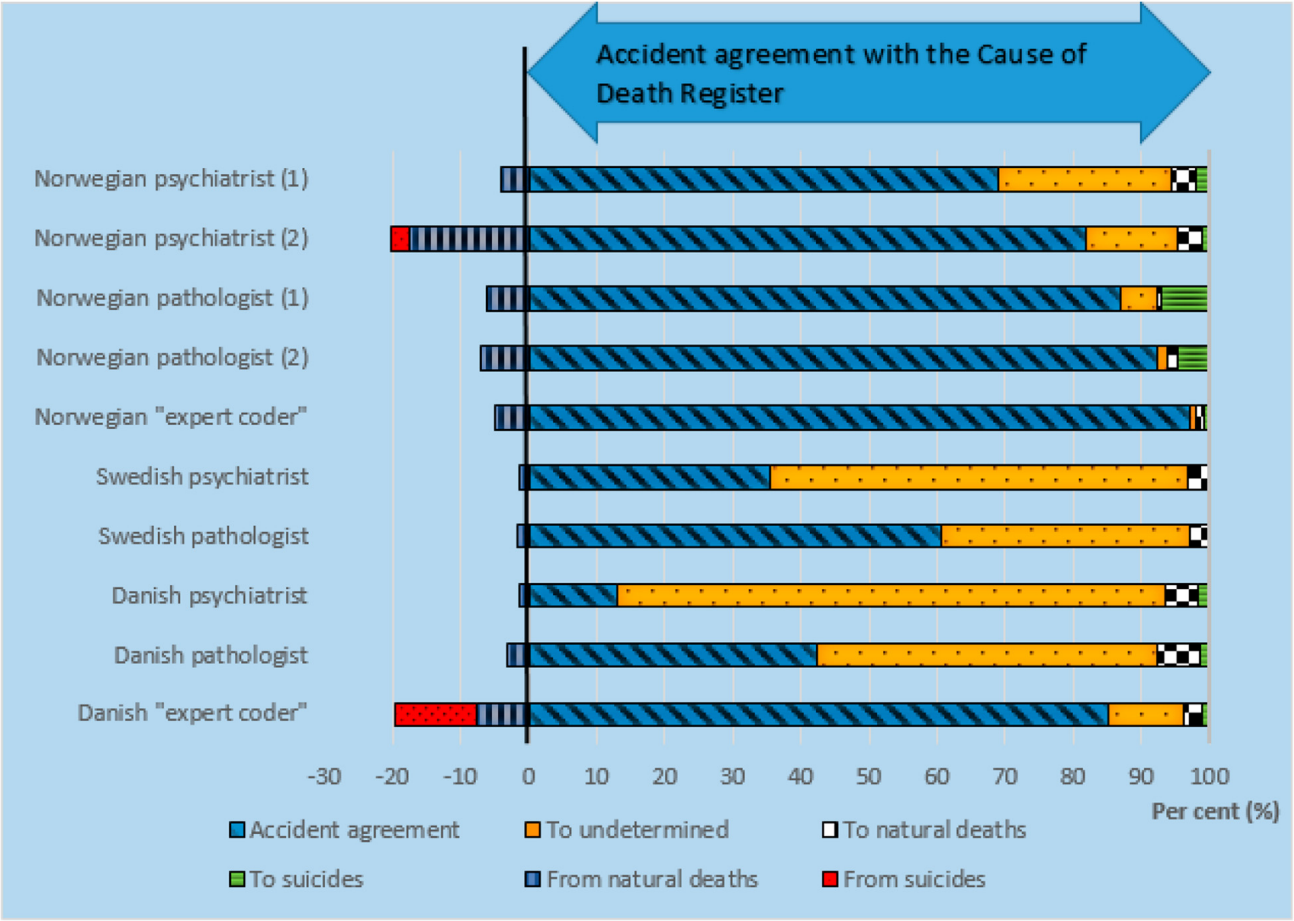
Reclassification of accidental deaths in the Norwegian dataset. First reclassification (1), and second reclassification (2) of the Norwegian cases. Agreement (*blue slanted lines*) in classification of manner of death between the Norwegian Cause of Death Register and the experts’. Bars to the left of the vertical *black line* shows the experts’ reclassification (in per cent) from accidents to undetermined, natural deaths and suicides. The bars to the right of the vertical *black line* shows the experts’ reclassifications (in per cent) of suicides and natural deaths to accidents

**Fig. 3:**
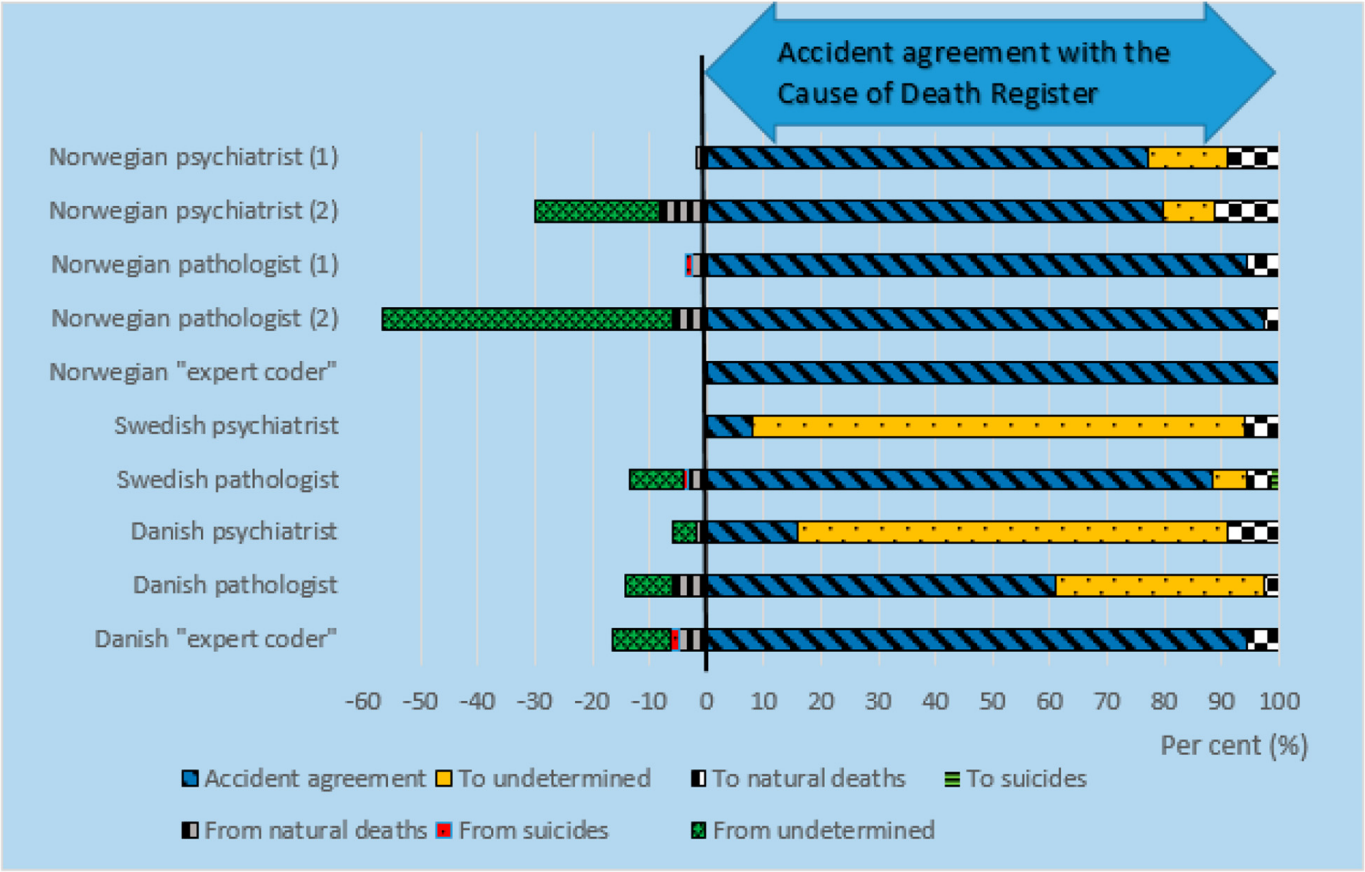
Reclassification of undetermined and accidental deaths in the Swedish dataset. First reclassification (1), and second reclassification (2) of the Swedish cases. Agreement (*blue slanted lines*) in classification of manner of death between the Swedish Cause of Death Register and the experts’ reclassifications. Bars to the left of the vertical *black line* show the experts’ reclassification (in per cent) from accidents to undetermined, natural deaths and suicides. The bars to the right of the vertical *black line* show the experts’ reclassifications (in per cent) of suicides, natural deaths and undetermined to accidents

**Fig. 4:**
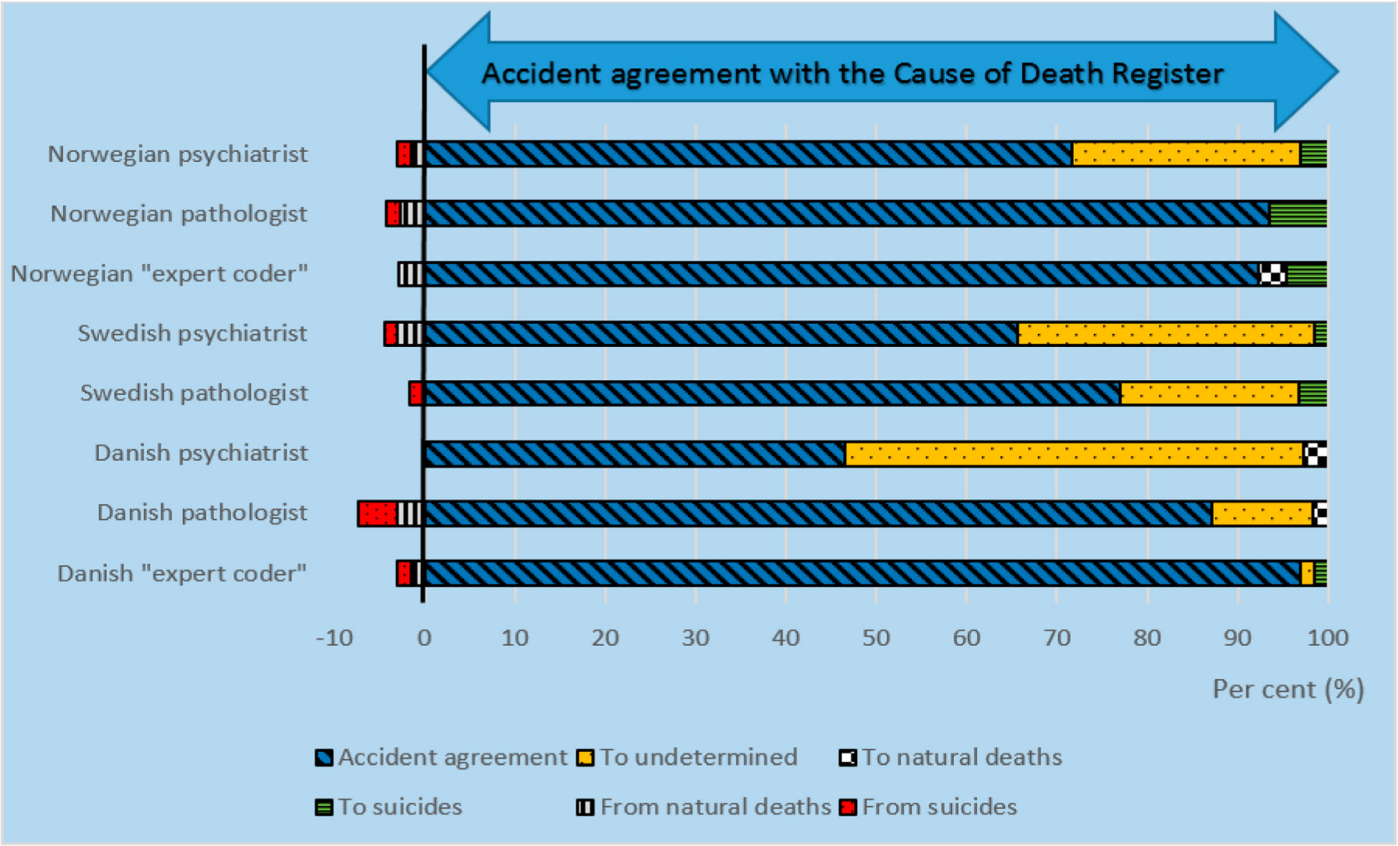
Reclassification of accidental deaths in the Danish dataset. Agreement (*blue slanted lines*) in classification of manner of death between the Danish Cause of Death Register and the experts’ reclassifications. Bars to the left of the vertical *black line* show the experts’ reclassification (in per cent) from accidents to undetermined, natural deaths and suicides. The bars to the right of the vertical *black line* show the experts’ reclassifications (in per cent) of suicides and natural deaths to accidents

There was 69 % (Norwegian and Swedish datasets) and 78 % (Danish dataset) agreement in classification of accidents between the official mortality statistics and the experts’ reclassifications (Table 3). In the majority of the disagreements, the experts reclassified accidents to undetermined manner of death: 26, 25 and 19 %, respectively (Figs. 2, 3 and 4). Few accidents were reclassified as suicides (0.5–2 %) or natural deaths (1–5 %). Among the accidental deaths, the agreement between the experts and the official statistics was the lowest in drowning and poisoning accidents. In the Swedish dataset, there was 95 % (range 83–100 %) agreement in reclassification of undetermined manner of deaths between the official statistics and the experts.

**Table 3:** Agreement in reclassification of accidents

Dataset	Accidents	Traffic accidents	Poisoning accidents	Drowning accidents	Accidental fire and flame
	%, (range)	%, (range)	%, (range)	%, (range)	%, (range)
Norwegian	69 (13–97)	83 (57–100)	65 (6–97)	49 (0–100)	86 (25–100)
Swedish	69 (8–97)	83 (14–100)	64 (11–89)	64 (0–100)	67 (0–100)
Danish	78 (47–97)	87 (61–100)	78 (32–100)	48 (22–78)	84 (62–100)

Agreement (in per cent) between the manner of death recorded in the national cause of death registers and the experts’ assessment. Ranges are given in parentheses

In the second re-evaluation of the Norwegian and Swedish datasets, both experts increased their agreement regarding accidents with the official mortality statistics from 76 to 87 % in the Norwegian dataset, and from 85 to 87 % in the Swedish dataset. The two experts increased their agreement regarding poisoning accidental deaths in the second re-evaluation. Among the accidents, the two experts agreed to the least extent with the official mortality statistics in the reclassification of drowning accidents. Of natural deaths in the Norwegian dataset, 13 % were reclassified as accidents. Of undetermined manners of death in the Swedish dataset, agreement decreased regarding undetermined manner of death (42 % agreement), and they reclassified 17 and 25 % as suicides, and 22 and 51 % as accidents.

### Level of certainty in classification

The level of certainty varied between the experts’ profession and country as well as between different accident categories (Table 4). Among the accidents, the experts reported insufficient information in a large proportion of the drowning accidents and undetermined manner of death. Overall, the experts were most uncertain about drowning and poisoning accidents.

**Table 4:** Level of certainty in the reclassifications

	Certain	Uncertain	Insufficient information
	n (%)	n (%)	n (%)
Norwegian dataset			
Transport accidents	86 (78 %)	10 (9 %)	14 (13 %)
Accidental poisonings	372 (70 %)	60 (11 %)	104 (19 %)
Accidental drownings	31 (49 %)	4 (6 %)	28 (45 %)
Accidental fire and flame	80 (84 %)	3 (3 %)	12 (13 %)
Swedish dataset			
Transport accidents	65 (69 %)	3 (3 %)	26 (28 %)
Accidental poisonings	141 (68 %)	22 (10 %)	45 (22 %)
Accidental drownings	33 (59 %)	7 (12 %)	16 (29 %)
Accidental fire and flame	32 (75 %)	1 (2 %)	10 (23 %)
Event of undetermined intent	68 (40 %)	12 (7 %)	90 (53 %)
Danish dataset			
Transport accidents	105 (86 %)	4 (3 %)	13 (11 %)
Accidental poisonings	204 (75 %)	26 (10 %)	41 (15 %)
Accidental drownings	28 (56 %)	11 (22 %)	11 (22 %)
Accidental fire and flame	63 (77 %)	9 (11 %)	10 (12 %)

The experts’ assessment of level of certainty (i.e., certain, uncertain and insufficient information) in determining manner and cause of death in the first re-evaluation. Cases that were classified as accidents or undetermined manner of death in the cause of death registers in the Norwegian, Swedish, and Danish datasets

In the second re-evaluation of the Norwegian dataset, the two experts reported the highest proportion of insufficient information for traffic (22 %) and drowning accidents (20 %), and most uncertainty for poisoning accidents (5 %). In the Swedish dataset, the two experts reported the highest proportion of insufficient information for undetermined manner of death (30 %) and drowning accidents (18 %), and most uncertainty for poisoning accidents (9 %) and undetermined manner of death (8 %).

### Inter-rater agreement between experts with similar professional background

Cohen’s *K* was calculated to determine the agreement between the experts’ reclassifications of manner of death. There was moderate agreement between the two expert coders’ reclassifications of the Norwegian dataset, *K* = 0.67, *p* < 0.001, 95 % CI [0.63, 0.71]. In the Swedish and Danish datasets there was very good agreement between the two expert coders, *K* = 0.92, *p* < 0.001, 95 % CI [0.89, 0.94] and *K* = 0.93, *p* < 0.001, 95 % CI [0.89, 0.96], respectively. For the psychiatrists, the *K*-values were in the ranges 0.33–0.55, 0.26–0.36 and 0.37–0.53 for the Norwegian, Swedish and Danish datasets, respectively. For the forensic pathologists, the *K*-values were in the ranges 0.40–0.52, 0.47–0.79 and 0.55–0.72 for the Norwegian, Swedish and Danish datasets, respectively.

## Discussion

This study was based on a reclassification of 1799 deaths among adults in the three Scandinavian countries. Furthermore, we studied all cases of accident and undetermined manner of death in the datasets, a total of 599 individuals, with regard to certainty in classification and possible “missed” suicides. The reclassification was performed by eight experts, and there was a large and real variation between the eight experts’ reclassifications. This expresses the level of uncertainty in comparing manner of deaths among the extracted categories of manners and causes of deaths.

Physicians often consider suicide when the method used is shooting or hanging and when the deceased leaves a suicide note; other scenarios might be more difficult to classify. Psychiatric disorder in general is a known risk factor for suicide [14, 15], and substance abuse is associated with higher risk of suicide [16]. A cross-sectional multicentre study of all admissions to hospital caused by self-poisoning in Oslo, Norway found that those hospitalized following suicide attempts more often had previous suicide attempts and reported more psychiatric treatment than those hospitalized for substance use [17]. An autopsy study among illicit drug users in Norway found that polydrug use was found in all manners of death (i.e., accident, suicide or homicide), but both the number and type of substances varied among the different manners of death [18]. Suicidal poisoning deaths had the lowest number of illicit drugs, but the highest total number of substances, with a high prevalence of anti-depressants and anti-psychotics [18]. In a register-based study from Sweden [19], previous psychiatric hospitalization was more common among suicides than undetermined manner of deaths, but hospitalization for substance abuse was more common among undetermined manner of death. In a psychological autopsy study from Utah, USA, decedents categorized as either undetermined manner of death or accidents were very similar in most variables, but on many key indicators of suicide risk, such as history of mental illness and/or psychiatric symptoms, decedents categorized as either undetermined, accident or suicide were similar [20]. This illustrates the difficulties in classification, especially among natural deaths with a psychiatric disorder, suicides, accidents and undetermined manner of death; the variations between the experts’ reclassifications in the present study illustrate this uncertainty.

The experts reported most uncertainty for drowning and poisoning accidental deaths in the first re-evaluation, and poisoning accidental deaths in the second re-evaluation when the experts had more information from the forensic autopsy including toxicological analyses. A large proportion of poisoning victims had been autopsied, but despite this high proportion of autopsies, the experts were most uncertain in poisoning accidents, implying the difficulties in determining the intention in poisoning accidental deaths. The two experts increased their agreement among poisoning accidental deaths in the second re-evaluation, implying the importance of a forensic autopsy including toxicological analyses. Among the extracted natural deaths in the Norwegian dataset, 13 % were reclassified as accidents in the second re-evaluation. A large proportion of the death certificates on natural deaths had sparse information, in many cases only “sudden death” and a psychiatric diagnosis, mostly psychoactive substance use. In a small proportion of natural deaths, a forensic autopsy had been performed (16 %). This low autopsy rate together with the sparse information on the death certificate makes the cause of death among sudden natural deaths uncertain. Whether a higher autopsy rate would reveal more accidents and suicides is unclear. In a retrospective autopsy study from Norway [21], 10 pre-autopsy death certificates reported alcohol dependence syndrome as the underlying cause of death, and in nine of these, the underlying cause of death was changed after hospital autopsy, i.e., changed to cirrhosis, cardiomyopathy and non-alcohol-related causes. The low forensic autopsy rates in Norway and Denmark may be explained by legislation: once homicide has been ruled out, the police may be less prone to seek further information about the cause of death. In Denmark, a forensic autopsy is compulsory if a criminal offence is suspected, the manner of death is unknown and the deceased has a known history of substance misuse [22]. In Norway, a forensic autopsy is compulsory if a criminal offence is suspected or if the identity of the deceased is unknown [23]. For all other deceased, the decision about whether to perform a forensic autopsy is taken by the different police authorities [24].

The combination of the medical history, an investigation on site and an autopsy is still considered the gold standard for determining the cause and manner of death. In all Scandinavian countries, both clinical and forensic autopsy rates have declined during the past 30 years, with forensic autopsy rates (number of autopsies divided by the total number of deaths in a particular region per year) of 5.8 % (Sweden), 4.1 % (Norway) and 2.3 % (Denmark) in 2010 [25–27]. Autopsies are important for quality control, teaching and legal protection, and autopsies can also reveal incidental findings with implications for both the patient’s family and the community. Autopsies are also important for obtaining more valid mortality statistics. A systematic review determined a median rate of 23.5 % (range, 4.1–49.8 %) of autopsies that detected important clinically missed diagnoses [28]. In a study from Norway, which retrospectively reviewed discrepancies between pre-autopsy cause of death and the hospital autopsy-derived cause of death, the autopsy led to a major revision of the underlying cause of death in 32 % of the cases (i.e., change in ICD chapter) [21]. In two autopsy studies from Denmark, the cause of death was found to be different in about one-third of the cases after autopsy [29], and the manner of death was changed in about 4 % of the cases [30]. Even when autopsies are made, however, there is still inherent uncertainty with autopsy such that even experts cannot always agree. The present study provides data on the level of disagreement between professionals and between countries.

In the present study, the majority of the extracted accidental deaths were also reclassified as accidents. Some accidents were reclassified as undetermined manner of death, and it is likely that some of these were suicides. In the Swedish dataset, most undetermined manner of deaths were reclassified as accidents, and about 20 % as suicides. About 300–400 deaths are classified as undetermined each year in Sweden, and if 20 % of these were suicides, the suicide rate would increase to a very small extent. Few accidents were reclassified as suicides, and the present study does not indicate a major under-reporting of suicides. There is some uncertainty in some of the classifications because of the limited information on the deceased, and this should be studied further.

Other studies have examined under-reporting of suicide, and have reviewed cases within one or a few categories of manner or cause of death in which suicides could be hidden (e.g., drowning accidents, poisoning accidents, traffic accidents, undetermined intent) [6, 31–33], a nationwide sample within a selected group (e.g., military deaths) [1, 34] or a sample from a selected region within a country [2–5, 35–39]. These studies came to very different conclusions, from high accuracy to substantial under-reporting. These different conclusions can be explained by the different included categories of manner and cause of death, differences between countries and studies from different periods of time (published between 1963 and 1999). The three review studies from the Scandinavian countries concluded with less than 10 % under-reporting of suicides [31, 32, 34]. The study of all military deaths in the USA reviewed deaths reported as undetermined manner of death or accidents due to gun-shot, overdose, drowning, falls and asphyxia to evaluate whether suicides were under-reported [1]. The authors found 17 % more suicides than reported, and an additional 4 % of deaths where they suspected suicide.

There was a real difference between the experts’ reclassifications, and accidents were more often reclassified as undetermined deaths by psychiatrists than by the other experts. The Norwegian expert coder used the WHO’s ICD coding manual systematically, while the other experts used more “clinical judgement”, which may explain the differences between the experts’ assessment in level of certainty. The re-evaluations of accidental deaths by the Norwegian expert coder were close to the official statistics in Norway (97 %) and Sweden (100 %), but differed more from those in Denmark (92 %). The Danish expert coder agreed with the official statistics in Denmark in 97 % of the accidental deaths, and 85 and 94 % in Norway and Sweden, respectively. There was very good agreement (Cohen’s *K*) between the expert coders regarding the Swedish and Danish datasets, and moderate agreement regarding the Norwegian dataset, which implies that the classifications in the cause of death registers are of good quality.

### Strengths and limitations of the present study

In the present study, a large proportion of cases within the extracted categories were included, where both genders are represented in these nationwide samples of adults, thus making the material comparable with the official statistics. The very different autopsy percentages in the three datasets are consistent with official data [13–15], in which Sweden has the highest and Denmark the lowest autopsy rate, and hence this consistency is a strength. The internal validity is considered quite good. The information given to the experts in the first re-evaluation was identical to the information used by the cause of death registers in Scandinavia, while the information given in the second re-evaluation was more comprehensive than the information used by the cause of death registers. We consider the second re-evaluation strengthened the present study as it supports the possibility that there may be some additional “hidden suicides”. The various included manners and causes of death, which are reported as categories of possible missed suicides in other studies [20], are an important strength when generalizing to the whole population. Another strength is that the samples were individually re-evaluated by eight persons from all three countries with different but relevant fields of expertise. There was no need for translation because of the quite similar languages across Scandinavia.

A methodological limitation of the present study, as well as a general challenge, is the major differences concerning the comprehensiveness of the information given on death certificates. In Norway and Sweden, death certificates contain sparse information, while Danish death certificates in cases of non-natural death present an excerpt of clinical information about the deceased, results of the death-scene investigation, information about the post-mortem examination, and selected information from an autopsy report where an autopsy was performed. These differences influenced the reclassification of the samples from the different Scandinavian countries. The low forensic autopsy percentage for non-natural deaths in the Norwegian and Danish datasets and the low autopsy frequency of the extracted categories of natural deaths, where there was limited information about the deceased, are both a methodological limitation and a general limitation of the mortality statistics.

In 2008, the Danish Cause of Death Register classified 115 deaths as undetermined intent (Y10-34, Y872) [40]. In the present study, undetermined intent was not included in the extracted Danish dataset, which might influence the results to some degree, with regard to a reclassification of undetermined manner of death to suicides and accidents.

## Conclusions

There was moderate agreement in reclassification of accidents between the official mortality statistics and the experts. In the majority of cases where there was disagreement, accidents were reclassified as undetermined manner of death, and only a small proportion as suicides. Despite the lowest autopsy rate in Denmark, there was highest agreement between the experts in the Danish data set. Denmark more often than Norway and Sweden perform external post-mortem examinations on site, and this information can in some cases be more useful than a forensic autopsy. This shows that the combination of more information both from more thorough external post-mortem examinations and an autopsy may provide more reliable mortality data.

## Abbreviations

ICD-10, 10th edition of the International Statistical Classification of Diseases and Related Health Problems; SNOMED, Systematized Nomenclature of Medicine; WHO, World Health Organization
